# Control of seminal fluid protein expression via regulatory hubs in *Drosophila melanogaster*

**DOI:** 10.1098/rspb.2018.1681

**Published:** 2018-09-26

**Authors:** Irina Mohorianu, Emily K. Fowler, Tamas Dalmay, Tracey Chapman

**Affiliations:** 1 School of Biological Sciences, University of East Anglia, Norwich Research Park, Norwich NR4 7TJ, UK; 2 School of Computing Sciences, University of East Anglia, Norwich Research Park, Norwich NR4 7TJ, UK

**Keywords:** gene regulatory network, regulatory hub, microRNA (miRNA), sexual conflict

## Abstract

Highly precise, yet flexible and responsive coordination of expression across groups of genes underpins the integrity of many vital functions. However, our understanding of gene regulatory networks (GRNs) is often hampered by the lack of experimentally tractable systems, by significant computational challenges derived from the large number of genes involved or from difficulties in the accurate identification and characterization of gene interactions. Here we used a tractable experimental system in which to study GRNs: the genes encoding the seminal fluid proteins that are transferred along with sperm (the ‘transferome’) in *Drosophila melanogaster* fruit flies. The products of transferome genes are core determinants of reproductive success and, to date, only transcription factors have been implicated in the modulation of their expression. Hence, as yet, we know nothing about the post-transcriptional mechanisms underlying the tight, responsive and precise regulation of this important gene set. We investigated this omission in the current study. We first used bioinformatics to identify potential regulatory motifs that linked the transferome genes in a putative interaction network. This predicted the presence of putative microRNA (miRNA) ‘hubs’. We then tested this prediction, that post-transcriptional regulation is important for the control of transferome genes, by knocking down miRNA expression in adult males. This abolished the ability of males to respond adaptively to the threat of sexual competition, indicating a regulatory role for miRNAs in the regulation of transferome function. Further bioinformatics analysis then identified candidate miRNAs as putative regulatory hubs and evidence for variation in the strength of miRNA regulation across the transferome gene set. The results revealed regulatory mechanisms that can underpin robust, precise and flexible regulation of multiple fitness-related genes. They also help to explain how males can adaptively modulate ejaculate composition.

## 1. Introduction

### (a) Gene regulatory networks

**Figure 1.**
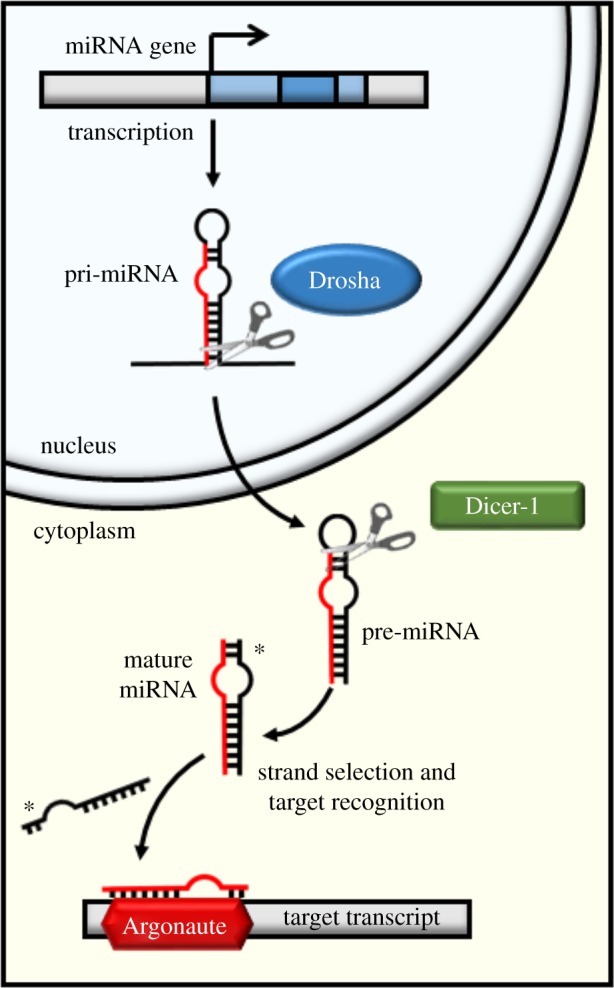
miRNA biogenesis. The miRNA biosynthesis pathway in *Drosophila melanogaster*, to indicate the Drosha manipulation applied in the empirical validation.

Genes rarely, if ever, function in isolation from one another. They are often interconnected within gene regulatory networks (GRNs) that regulate a specific pathway or function. Genes may be regulated at the transcriptional, or post-transcriptional levels. Transcription factors (TFs) control the rate of gene transcription by binding specific DNA motifs, usually upstream of the coding region [1]. Post-transcriptional regulation can be achieved by small RNAs (sRNAs), which target mRNA transcripts, inhibiting translation into proteins. One class of well-studied sRNAs are the 22 nt microRNAs (miRNAs) [2]. These are processed from a hairpin-like structure by Drosha and Dicer-1 enzymes (figure 1) and then loaded into the Argonaute protein, part of the RNA induced silencing complex (RISC), which guides the miRNA to the target mRNA [3]. In animals, miRNAs generally induce translational repression in their targets via matching of the miRNA ‘seed’ sequence (at positions 2–8 from the 5′ end) to the 3′ untranslated region (UTR) of the target mRNA [3,4]. Small interfering (si)RNAs (e.g. 21 nt siRNAs, repeat associated RNAs (rasiRNAs), promoter associated (pas)RNAs and 27–30 nt piwi associated (pi)RNAs) are processed by Dicer-2 and recruit different Ago proteins [5]. However, many details of their regulatory roles are not yet known [5].

Our knowledge of gene regulation is rapidly growing. However, the identification and comparison of inter-relationships between GRNs poses significant challenges [6]. For example, GRNs are often inferred from gene expression profiles, which may contain high and variable amounts of noise [7,8]. GRNs can also be identified by using protein–protein interactions [9,10], from steady state and manipulated datasets (e.g. knockouts) and from the integration of gene expression with metabolomic data [11]. GRNs range from simple to the very complex, comprising many hundreds of genes and TFs [12]. Valuable insights can be gained by identifying and comparing GRNs across different cells, tissues and over time [13–16]. For example, in evolutionary biology there is much interest in determining how core features of GRNs such as topology, composition, connectivity, robustness to mutation, clustering and stability change under selection [17–21]. A key, and so far unanswered question, is how selection acts in different environments to achieve network stability and indeed whether one can measure the degree of stability from core network features [19]. The general emerging idea is that genes that are components of a regulatory unit are likely to be linked or co-regulated through one or multiple ‘hubs’ or switches that are essential for network organization and hence themselves targets of selection. GRNs may also represent an efficient way to capture and maintain the effects of beneficial mutations, or to maintain selectively neutral ones [20].

A practical hurdle can be the difficulty in identifying an appropriate set of genes in which to study features of GRNs, both at the level of gene expression and the resulting phenotype. To facilitate the understanding of such a system, it should ideally: (i) comprise a tightly linked network of genes, (ii) represent a set of genes within a defined biological process and/or localized expression, (iii) be genetically tractable for experimental testing, and (iv) produce a well-defined and measureable phenotype. The set of genes that encode the non-sperm components of the ejaculate in male *Drosophila melanogaster* fruit flies [22] (hereafter the ‘transferome’ genes) fulfils these criteria. They represent a potentially valuable exemplar because they: (i) show coordinated expression [23–25], (ii) have defined functions and easily measureable phenotypes [26], and (iii) can be subjected to controlled, experimental genetic manipulations.

### (b) Functions and significance of the reproductive transferome

Seminal fluid proteins that comprise the transferome are of key importance across many animal taxa [22,27,28] and are far more than a buffer to maintain sperm osmotic potential [29,30]. In *D. melanogaster* these remarkable substances remodel female behaviour, physiology, gene expression and fitness [26,31]. Individual seminal fluid proteins affect egg production, sexual receptivity, feeding and nutrient balancing, sleep patterns, sperm retention and usage, water balance and antimicrobial peptide production (reviewed in [28]). These actions are fundamental to reproductive success [32–34]. Seminal fluid components in *D. melanogaster* have been well characterized at the genetic, functional and structural levels [27]. Isotopic 15 N labelling has defined a set of extracellular proteins secreted by the male accessory glands, ejaculatory ducts and bulb, plus non-sperm molecules from the testes that represent the transferome and that are transferred to females during mating [22].

### (c) The transferome as a gene regulatory network that responds to the socio-sexual context

Male *D. melanogaster* exposed to rivals prior to mating for at least 24 h mate for significantly longer and transfer more of key seminal fluid proteins into females [35]. Such responses are precise, robust and flexible [36,37] and result in significantly increased male fitness [38]. Hence ejaculate composition can be modified in a highly sophisticated manner in response to social and sexual context [35,39]. This is also underpinned by differential expression in transferome-encoding genes [40]. Together these data support the idea that males calibrate responses to sexual competition with remarkable precision and suggest that the transferome genes may be linked in a tight and highly coordinated regulation in response to the environment [40]. However, little is yet known about how this is achieved.

We hypothesize, and test here, that an effective way in which to regulate the many individual transferome components within a GRN is to manage them in ‘subsets’ controlled by the same regulators. This could facilitate rapid and coordinated expression of groups of genes when required. This level of control could be achieved by TFs that modulate the transcription of sets of genes, and/or by miRNAs that bind to mRNA transcripts and repress or ‘manage’ the translation of functionally linked groups of proteins [41]. Previously, transcriptional regulation of transferome genes has been reported, via known TFs (e.g. [42]). However, whether miRNAs are similarly involved to effect post-transcriptional regulation is not yet known, which provides the main motivation for this current study. We consider the transferome genes as a unit because males control the expression of these genes and appear to be able to alter the precise composition of ejaculate transferome proteins in order to effect variation in post-mating responses (PMRs) [39]. Hence males have the ability to control and coordinate the collective synthesis of transferome proteins. This will occur even if some individual transferome components are involved in mediating phenotypes subject to sexually antagonistic selection [32].

We adopted a predictive approach to test these ideas and focused on tests of potential post-transcriptional regulation by miRNAs. Many analyses of potential links between regulatory elements and their targets suffer from a high false positive rate [43]. We minimized this difficulty here by first analysing genomic data to investigate, via detection of putative miRNA binding motifs, whether there was any evidence for post-transcriptional regulation and hence any evidence for regulation by GRNs, in comparison to what would be expected by chance. We first tested whether we could identify known sequence motifs shared between members of the transferome gene set. We detected miRNA seed region motifs along the 3′ UTRs of all transferome genes in order to test for evidence of putative regulation by miRNAs. The results indicated the existence of miRNA regulatory ‘hubs’ with the capacity to control specific subsets of transferome genes. To directly test the prediction that miRNAs can influence the transferome phenotype, we then reduced miRNA expression in adult males by knocking down the gene encoding a major upstream component of the canonical miRNA synthesis pathway (Drosha). Females mating to *drosha* knockdown males exhibited reduced post-mating receptivity responses, supporting the hypothesis that miRNAs regulate transferome functions as a whole. Further bioinformatics analysis revealed evidence for specific candidate miRNAs as well as variation in the number/type of shared regulatory sequences. The results shed new light on how complex sets of gene products involved in key fitness-related functions can be managed.

## 2. Methods

Using the set of 136 genes encoding the *D. melanogaster* transferome proteins [22], we first scanned the 3′ UTR regions for miRNA seed sites, to test for evidence of co-regulation of transferome genes by miRNAs as a whole. This analysis provided evidence that transferome genes were enriched for some miRNA seed sequences. Hence, we then tested empirically, the hypothesis that miRNAs as a whole regulate the transferome phenotype, by knocking down miRNA biosynthesis. Having confirmed the role of miRNAs in transferome gene regulation, we then refined our bioinformatics analysis to identify general features of miRNA regulation as well as specific candidate miRNAs. All analyses were conducted at the transcript level and to account for the presence of different transcript isoforms partially sharing UTR sequences, we also generated gene-level results.

### (a) Regulation of transferome genes by known microRNAs

A conservation analysis was first conducted to identify all miRNAs in the *D. melanogaster* genome. All mature miRNAs from 12 *Drosophila* species [44] were mapped on the *D. melanogaster* genome and the miRNA loci then determined using criteria based upon the identification of miRNA hairpin-like secondary structures (specifically: adjusted minimal folding free energy (aMFE) < −20 and no branching adjacent to the miRNA/miRNA* duplex) [45]. We then determined all 7 and 8 nt seed regions for all mature miRNAs. miRNAs sharing seed regions (perfect identity) were collapsed under one entry. Seeds were mapped to the 3′ UTRs of the transferome gene transcripts (with full length matching and no mis-matches or gaps allowed). The enrichment of miRNA usage was calculated by comparing the number of target genes for each miRNA seed site, on the transferome transcripts and on all *D. melanogaster* transcripts, using identical targeting criteria for both analyses. We used the Fisher exact test to evaluate whether the observed number of putative targets was in line with the expectation across the *D. melanogaster* genome or whether it was enriched/depleted for transferome transcripts.

### (b) Empirical validation of effect of microRNA manipulation on the expression of the transferome

We conducted an empirical test of the hypothesis arising from the initial sequence analyses showing that miRNAs regulate the expression of transferome genes and hence the transferome phenotype itself. We tested the role of miRNA regulation in this process, using knockdown of *drosha*. We tested the collective role of miRNAs in the transferome phenotype, rather than tests of individual predicted miRNA hubs, because individual knockdowns of miRNAs may often yield undetectable effects on phenotypes [46] potentially owing to complex interactions, redundancies and feedback loops in the networks in which the individual miRNAs are embedded.

#### (i) General fly rearing and experimental procedures

We tested directly the effect of matings with males with reduced miRNA levels on female post-mating behaviour. To do this, *drosha* knockdown was restricted to the male accessory glands, the tissue in which the majority of the transferome proteins are synthesized. We first tested whether *drosha* knockdown males responded to the presence of rivals (and hence the threat of sperm competition) by subsequently mating for longer [38]. We then examined whether the knockdown of *drosha* impaired the ability of a male to respond to higher levels of sperm competition by reducing receptivity of his mate [38] via the transfer of an altered set of seminal fluid proteins to females [35,47].

Fly rearing and all experiments were conducted at 25°C, 50% humidity and a 12 : 12 h light dark cycle. Flies were reared throughout on sugar yeast agar food (100 g brewer's yeast, 50 g sugar, 20 g agar, 30 ml Nipagin (10% w/v solution) and 3 ml propionic acid per litre of medium). Experiments were conducted in glass vials (25 mm × 75 mm) containing 7 ml food medium. All flies were raised at standard larval density, and upon eclosion, adults were sex-separated under ice anaesthesia. For the mating assays, 2–3 day old focal males of each line were transferred to vials and housed either singly or with a ‘rival’ wild-type male for 4 days. Rival males were wing-clipped under CO_2_ anaesthesia for identification. Wild-type virgin females were kept in groups of 10 until the day before the experiment, and then housed singly. On the day of the experiment, single focal males were transferred into each female vial. Each pair was observed for 3 h, and mating times recorded. Immediately after mating, the male was discarded, and the female retained for 24 h, then individually transferred to new vials each containing a single wild-type male. Females were allowed a 3 h time-window to re-mate, and the total number of rematings were recorded.

#### (ii) Fly stocks

Wild-type flies were from the Dahomey stock used previously (e.g. [36,38]). *drosha* RNAi lines were obtained from the Vienna Drosophila Stock Centre (stock v108026). Males from these lines were crossed to female *Acp26Aa-P-Gal4* flies in which *Gal4* is driven under the direction of the *Acp26Aa* accessory gland main cell-specific promoter [48], to generate male offspring with an accessory-gland specific knockdown of *drosha*. Since the Gal4 is X-linked in the *Acp26Aa-P-Gal4* line, control lines lacking the *Gal4* driver were derived from the reciprocal cross.

#### (iii) RNA extractions and quantitative RT-PCR

50 pairs of accessory glands were dissected from four replicates of each line and pooled in phosphate buffer solution. The tissues were disrupted by grinding under liquid nitrogen and total RNA extracted (miRvana miRNA isolation kit, *Ambion*) according to the kit protocol. RNA was eluted in RNA storage solution (*Ambion*) and quantified using a NanoDrop 8000 (*ThermoScientific*). RNA preparations were treated with TURBO™ DNase using the TURBO™ DNA-free kit (*Ambion*), prior to reverse transcription to cDNA using the QuantiTect^®^ Reverse Transcription Kit (*Qiagen*). Quantitative RT-PCR (qRT-PCR) was used to verify knockdown in *drosha* transcript levels, using *CG13220* and *eIF-1A* as reference genes. Assays were run using a StepOnePlus™ machine (*Life Technologies*) and iTaq Universal SYBR^®^ Green Supermix (*Bio-Rad*). PCR cycling conditions were: 95°C for 30 s, followed by 40 cycles of 95°C for 15 s, and 60°C for 1 min. Melt curve analysis was carried out according to the default settings, and all samples showed a single product. Primers were optimized using serial dilutions of cDNA from 50 ng to 0.016 ng as template in a total reaction volume of 20 µl, with triplicate technical replicates. Efficiencies were between 90% and 105%. For verification of experimental samples, cDNA from 10 ng total RNA was used in each 20 µl reaction. The primer sequences were:

*drosha* 5′ AGATGCCAGAGAACTTCACCATCCA, 5′ GAAAGAAGTGAAAAGCTGGGCAGGA; *CG13220* 5′ TGGTGAGCTACGGAGCCCTTG, 5′ GGGGCCTGCCGTAAATGTAGA;

*eIF-1A* 5′ ATCAGCTCCGAGGATGACGC, 5′ GCCGAGACAGACGTTCCAGA.

### (c) Statistical analyses

Statistical analyses of the phenotypic data were performed using custom scripts in R v. 3.1.2 [49]. Comparisons were made between treatments (rival/no rival) within each line (mutant/control). Mating duration data were not normally distributed (Shapiro–Wilk, *p* < 0.05) and were compared using Wilcox–Mann–Whitney *U* tests. Total numbers of re-mating females were analysed using χ^2^ tests.

## 3. Results and discussion

The initial bioinformatic results showed significant over-representation among transferome genes of 37 specific miRNA seed sequences along with global under-representation (in comparison to binding sites present in all 3′ UTRs) of miRNA binding sites among the transferome set as a whole. This indicated a pattern of multiple transferome genes sharing the same miRNA seed sequences—thus potentially subject to tight, coordinated control by a few miRNA hubs. This was confirmed by the empirical tests, showing that the transferome phenotype was significantly altered when miRNAs were globally reduced. The extensive additional bioinformatics analysis highlighted specific candidate miRNAs as regulatory hubs in the control of the transferome genes.

### (a) Global regulation of transferome genes by microRNAs

We first evaluated the over-representation of miRNA target sites among the 3′ UTRs of the transferome genes (approx. 1500 transcripts), when compared to the entire set of *D. melanogaster* 3′ UTRs. The transcript-level data and enrichment for the miRNA analysis are shown in the electronic supplementary material, table S1a,b,c and the gene-level data in the electronic supplementary material, table S1d,e,f. We found 37 miRNAs whose targets were significantly enriched among transferome transcripts (electronic supplementary material, table S1c). The most significantly enriched target site was that of miR-4943-5p, which has seed sites in 80 transferome 3′ UTRs (corresponding to 42 genes). In contrast to the typical pattern of miRNA biogenesis, the miR-4943 locus spans the sense strand of an exon/intron boundary in the gene *CG5953*, rather than from an intronic or intergenic region. Interestingly, this miRNA appears to be lineage-specific (i.e. restricted to *D. melanogaster*) and expressed at relatively low levels [50]. In total, the targeted 3′ UTRs of all enriched miRNAs corresponded to 71 genes, approximately half of the transferome set. We observed no particular functional enrichment for this subset of 71 genes. Having predicted significant enrichment for miRNA seed sequences among transferome genes, we then tested experimentally, as described in the next section below, whether there was empirical evidence that knockdown of miRNAs as a whole altered the transferome phenotype.

### (b) Empirical validation of effect of microRNA manipulation on the expression of the transferome

**Figure 2.**
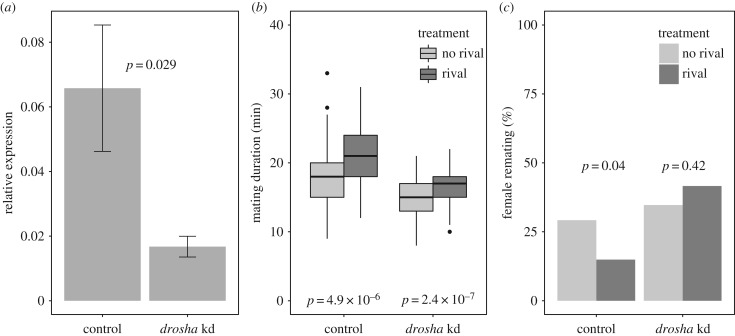
Manipulations to miRNA biosynthesis abolishes the ability of males to alter ejaculate composition adaptively. (*a*) Significant knockdown of *drosha* RNA in male accessory glands (qRT-PCR; relative expression normalized against *CG13220* & *eIF-1A*). The gene for Drosha was individually targeted for knockdown in male accessory glands using main cell promotor-specific GAL4 (Acp26Aa-P-Gal4) to drive the expression of UAS-*drosha*-IR (inverted repeat), to result in RNA interference of *drosha* transcripts. Control males generated for each line were from the same genetic background as the knockdowns, but lacked the GAL4 driver. (*b*) Significant extension to mating duration retained in control and *drosha* knockdown males following exposure to rivals: control, *p* = 4.9 × 10^−6^, *n* (rivals)=77, *n* (no rivals) = 96; *drosha* kd, *p* = 2.4 × 10^−7^, *n* (rivals) = 90, *n* (no rivals) = 97. (*c*) Loss of ability of *drosha* knockdown ejaculates (ns) to reduce female receptivity following exposure to rivals, response retained in controls (control, *p* = 0.04, *n* (rivals) = 74, *n* (no rivals) = 96; *drosha* kd, *p* = 0.42, *n* (rivals) = 89, *n* (no rivals) = 95).

We tested the prediction from the initial bioinformatics analyses above, that miRNAs play an important role in the global regulation of PMR genes. We did this directly by measuring the phenotypic effect of miRNA reduction in males on the post-mating behaviour of their mates. The knockdown manipulation was effective and a significant reduction in *drosha* expression was achieved (figure 2*a*; Wilcox–Mann–Whitney *U* test, *p* = 0.029). Mating durations in both control and *drosha* knockdown lines were significantly longer when males had previously been kept with a rival in comparison to individually housed (figure 2*b*; Wilcox–Mann–Whitney *U* tests, control: *p* = 4.9 × 10^−6^; *drosha* knockdown: *p* = 2.4 × 10^−7^). Therefore, reducing *drosha* expression in accessory glands had no effect on the ability of males to detect the presence of rival males, or to alter their mating behaviour in response. Females previously mated to knockdown and control males, under rival and no-rival conditions, were given an opportunity to re-mate with a wild-type male after 24 h. For *drosha* controls, we found significantly fewer re-matings occurred in males previously exposed to rivals, as observed in wild-type flies. However, this effect was absent in the *drosha* knockdown males, in which there was no significant difference in numbers of re-matings between the rival and no-rival treatments (figure 2; χ*^2^* test: control, *p* = 0.04; *drosha* knockdown, *p* = 0.42). Hence, the knockdown of *drosha* significantly impaired the ability of males to decrease sexual receptivity of their mates, following exposure to rivals. This provides evidence that these males transferred an altered composition of seminal fluid proteins, specifically in terms of its receptivity-inhibiting properties, which may reflect the importance of miRNAs as regulators of this process [48,51]. Overall, the manipulations of miRNA biosynthesis by *drosha* knockdown validated the prediction that miRNAs regulate the functions of transferome genes.

Pleiotropic effects of *drosha* silencing were minimized as knockdown was restricted to the accessory glands of adult males. Knockdown males showed normal mating behaviour and extended mating duration responses to rivals. Hence, our evidence suggests that the re-mating receptivity effect on females was indeed modified by variation in the composition of the ejaculate that males transferred, rather than by pleiotropic effects of mating behaviour itself.

### (c) Identification of candidate microRNA regulatory hubs and variation in the extent of microRNA-mediated regulation

**Figure 3.**
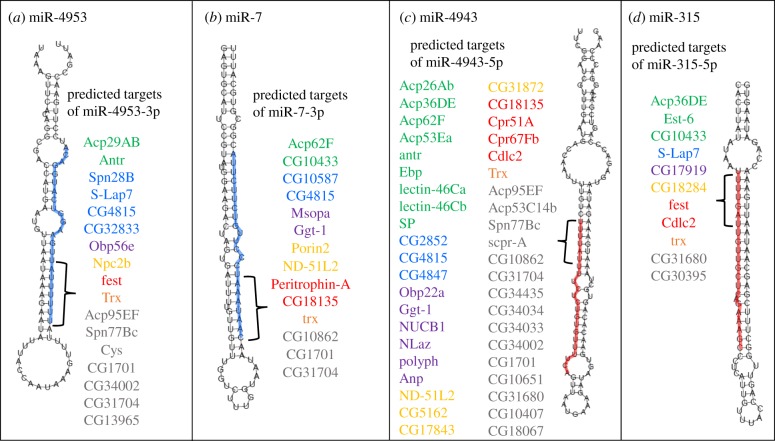
Four exemplar miRNAs as putative regulatory hubs. Secondary structures of four miRNAs with 100% complementarity between the 7-mer seed region (indicated by curly brackets) and the 3′ UTRs of multiple transferome-encoding genes. (*a*) The 7-mer seed region of miR-4953-3p has matches to the 3′ UTR of 17 genes. (*b*) The seed-site of miR-7-3p matches 14 genes. (*c*) The seed site of miR-4943-5p matches 42 genes. (*d*) The seed-site of miR-315-5p matches 11 genes. The gene names are coloured according to broad functional categories: post-mating behaviour/response (green); protein processing (blue); response to stimulus (purple); metabolic process (yellow); development/cellular organization (red); gene expression (orange); unknown function (grey).

We next conducted more extensive bioinformatics analysis to explore the presence of specific miRNA seed sites among transferome genes, regardless of any enrichment in comparison to the entire genome. We present the predicted target genes of each known miRNA (electronic supplementary material, table S1d,e) and the number and identity of miRNA seed sites on every transferome 3′ UTR (electronic supplementary material, table S1f). The interactions between miRNAs that can target the transferome genes and their corresponding targets are presented as a Cytoscape network diagram [52] (electronic supplementary material, figure S1). It is clear from the node sizes that the majority of known miRNAs target very few transferome genes. Indeed, 213 miRNAs had only 1–2 seed sites among all transferome 3′ UTRs. However, it was also apparent that some miRNAs have putative target sites in many different genes, and have the potential to act as regulatory ‘hubs’, simultaneously controlling many different genes. The miRNAs with the highest number of predicted target genes were miR-4943-5p (42 genes), miR-4953-3p (17 genes), miR-7-3p (14 genes), miR-315-5p (11 genes) and miR-9369-3p (10 genes) (figure 3).

To investigate if the genes targeted by the same miRNA shared functional profiles, we performed a gene ontology (GO) enrichment analysis on groups of ≥ 10 genes, using the list of 136 transferome genes as a reference set (electronic supplementary material, table S2; g:Profiler http://biit.cs.ut.ee/gprofiler/ [53]). We found no GO enrichment of terms for the targets of miR-4943, miR-4953 or miR-9369. However, significant enrichment of some biological process terms was found for miR-7 and miR-315 targets. Putative miR-7 targets were enriched for ‘organonitrogen compound metabolic process’, which characterized nine of the 14 genes (*Acp62F*, *trithorax*, *Peritrophin-A*, *ND-51L2*, *Ggt-1*, *CG10862*, *CG10585*, *CG31704*, and *CG4815*). The products of these genes are all predicted to be involved with protein processing (e.g. proteases, protease inhibitors, histone modification and chitin binding). For miR-315 targets, three of 11 genes were associated with ‘nervous system development’—*wurstfest*, *trithorax*, and *Esterase*-6. The products of these genes have diverse functions in translational and transcriptional control and pheromone processing [54–56].

To test whether the sets of genes putatively co-regulated by the same miRNAs belonged to the same gene families, we evaluated similarity between UTRs predicted to be regulated by the same miRNA hubs. For each regulatory feature (i.e. miRNA seed) we calculated the sequence identity using Clustal Omega, reported as a proportion of the length of the shortest transcript in each case. For the majority of UTRs, for each putative miRNA regulatory hub, the maximum transcript similarity was less than 60% (electronic supplementary material, figure S2), indicating low similarity between the targets and little evidence that the genes targeted by the same hubs were paralogues. This is consistent with the idea that the targeted genes can be unrelated, but owing to shared or coordinated biological functions they may have independently acquired shared regulatory motifs, allowing them to be controlled simultaneously. Further investigation of the patterns of the evolutionary acquisition of regulatory motifs across paralogous versus unrelated transferome genes would be useful to investigate this further, as well as knowledge of the detailed patterns of evolutionary change across the whole of the UTR regions.

Of the 136 transferome genes, 104 had at least one putative miRNA target site on their 3′ UTRs. The genes with the highest number of miRNA target sites were *trithorax* (putative sites for 50 miRNAs) and *wurstfest* (putative sites for 42 miRNAs). As these genes encode transcriptional and translational regulators, respectively, they may also require tight regulation themselves. Indeed, there is evidence from *Drosophila* [57], and from mice, that genes whose products are involved in a regulatory role (such as TFs) have more predicted miRNA target sites in their 3′ UTRs than housekeeping or structural genes [58]. Another nine genes were predicted to have more than 15 binding sites corresponding to different miRNAs. Among those genes were three whose products potentially play a role in cell development—*CG18135* which is known to interact with the unconventional myosin Myo10A [59], *CG10433*, which when over-expressed in male flies leads to defective microtubule organization [60], and *β-tubulin at 85D* which has been shown to regulate salivary gland migration [61]. Another two genes, *polyphemus* and *Niemann–Pick type C2b*, encode products involved in the immune response [62,63]. The remaining four genes with more than 15 miRNA sites have no experimentally confirmed functions, but may be involved in chitin-binding (*Peritrophin-A*), calcium ion binding (*regucalcin*) and protein-folding (*CG2852*). *CG18067* encodes a protein of unknown function.

**Figure 4.**
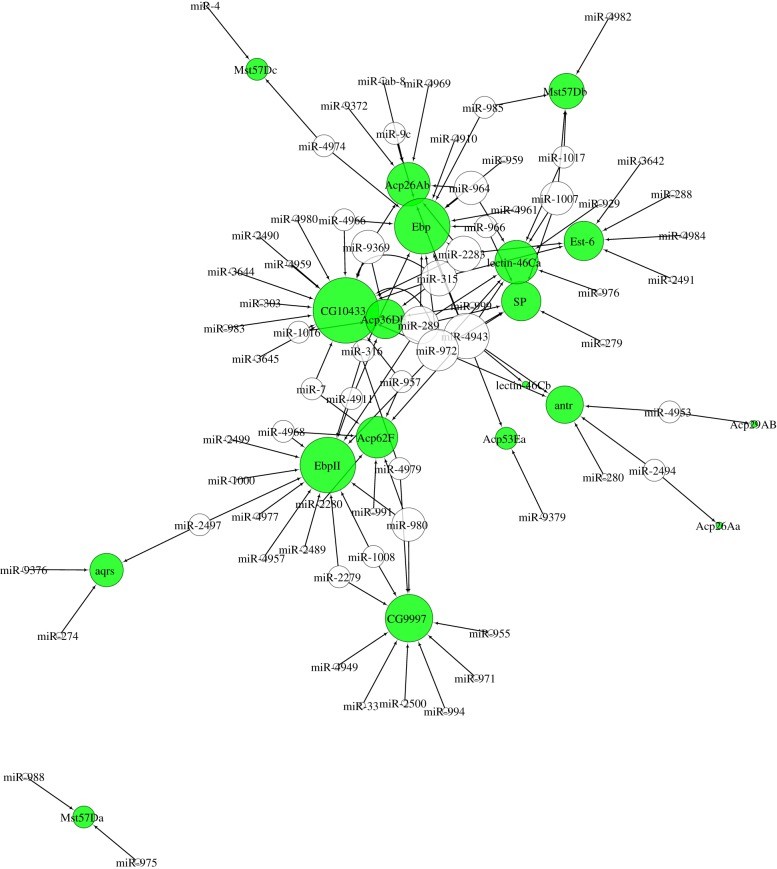
miRNA regulation of the post-mating response (PMR). Network of putative interactions between miRNAs (colourless nodes) and transferome genes (green nodes) whose products have a function in sperm storage and the PMR of females. The size of the node is proportional to the number of edges.

To gain further insight into whether a subset of genes, whose products are involved in similar biological processes, could be regulated by miRNA ‘hubs’, we created a Cytoscape network diagram [52] of 19 female PMR genes (figure 4). We know that ejaculate proteins that affect sperm storage and female behaviour are precisely controlled by the male fly in response to sperm competition, so we reasoned that these genes may be co-regulated by the same miRNAs. As for the entire transferome gene set, the most prolific miRNA among the PMR subset was miRNA-4943. Of the 19 genes chosen, nine had target sites for miR-4943 (*Acp26Ab*, *Acp36DE*, *Acp53Ea*, *Acp62F*, *antr*, *Ebp*, *lectin-46Ca*, *lectin-46Cb*, and *SP*). Although the term ‘post-mating behaviour’ was not significantly enriched in the GO analyses of miR-4943 targets described above, the fact that almost half of the PMR subset have miR-4943 target sites suggests that this miRNA may regulate sperm storage and PMR genes. Other potential PMR regulators were miR-972 and miR-289, which both had complementarity to *CG10433, Ebp, EbpII, lectin-46Ca*, and *SP*. miR-972 was also predicted to bind *antr*. It is also apparent (figure 4) that some PMR genes have target sites for an abundance of different miRNAs (e.g. *CG10433*, *Ebp* and *EbpII*), and thus instead of being regulated by a single ‘hub’, these genes may require very tight control, mediated by many different regulators.

Overall, our results indicate that several candidate miRNAs are predicted to regulate multiple transferome genes, thereby acting as regulatory ‘hubs’. Groups of genes with seed sites for the same miRNA are not necessarily enriched for a particular function, suggesting that their coordinated regulation impacts on diverse reproductive processes. In addition, we observed considerable redundancy in miRNA seed sites for individual genes, i.e. genes with seed sites corresponding to numerous different miRNAs. This suggests that some transferome genes may require particularly tight regulation, potentially because they themselves are transcriptional or translational regulators [58].

A key step for future studies would be to test the effect of removing the individual candidate miRNAs predicted by the in depth bioinformatics analyses we have conducted here. However, our initial investigations and the work of others suggest that this may be empirically challenging, owing to technical difficulties in achieving effective knockdown of single miRNAs [46] due to redundancy and feedback loops between the multiple regulatory elements involved. This very redundancy may itself be an important characteristic of such systems, contributing to their robustness.

## 4. Conclusion

The results showed evidence for the presence of regulatory miRNAs that modulate the expression of seminal fluid transferome genes in *D. melanogaster*, and more broadly, that cross referencing of regulatory regions to existing databases and unbiased methods for detecting regulation of unknown origin can successfully reveal signatures of gene regulation. We found significant over-representation of specific miRNA seeds and global under-representation of miRNA binding sites. This predicted that miRNAs regulate the expression of transferome genes, and was confirmed using knockdown of miRNA biosynthesis in males, which altered the expression of the transferome phenotype. Interestingly, several miRNAs were predicted as putative regulatory hubs, with seed sequences mapping to multiple transferome genes. There was also variation in the extent of seed mapping to transferome genes, suggesting some transferome genes are more tightly regulated than others. The observed variation in number or type of regulatory interactions would be interesting to study further as well as the potential fitness benefits of multiple layers of regulatory control, via the manipulation of individual regulatory components. Layers of gene regulation mediated by miRNAs as well as known TFs [42] could facilitate a robust and precise response across multiple, diverse genes. The next steps are to test this hypothesis experimentally on a genome-wide scale and to determine whether this is an emergent property of efficient GRNs. Whether there is any functional significance to the potential for regulation by miRNAs as well as other regulatory elements such as TFs will also be important to resolve.

Our results are especially interesting given the complexity of the transferome phenotype, with some key seminal fluid proteins contributing multiple phenotypic effects and others not. It has recently been suggested that this complexity itself is maintained by sexually antagonistic interactions between the sexes (e.g. over how much to invest in reproduction now versus later, etc.) [64]. Such complexity may also confer benefits to males in slowing the evolution of resistance to transferome effects in females [64]. The potential for precise post-transcriptional regulation of whole sets of transferome genes by miRNAs that we have uncovered here provides a mechanism by which males could adaptively modulate the composition of their ejaculates. Whether individual males have the potential to do this within or across different matings will be interesting to investigate. Overall, the results contribute to the growing realization of the fascinating level of sophistication underlying male–female interactions.

## Supplementary Material

Tables S1, S2

## Supplementary Material

Figures S1, S2

## Data accessibility

All sequence data are already public. Additional raw data for the phenotypic assays are archived in the Dryad Digital Repository: http://dx.doi.org/10.5061/dryad.fm15b38 [65].
