# Supplementary material for: Control of seminal fluid protein expression via regulatory hubs in Drosophila melanogaster
Source: Proc Biol Sci. 2018 Sep 26;285(1887):20181681. doi: 10.1098/rspb.2018.1681 (PMC6170815; doi:10.1098/rspb.2018.1681)
Supplement: Figures S1, S2 [file rspb20181681supp2.pdf]

Control of seminal fluid protein expression via regulatory hubs in *D. melanogaster*

Irina Mohorianu[1,2*], Emily Fowler[1*], Tamas Dalmay[1], Tracey Chapman[1,#]

[1]School of Biological Sciences, University of East Anglia, Norwich Research Park, Norwich, NR4 7TJ, UK.

[2]School of Computing Sciences, University of East Anglia, Norwich Research Park, Norwich, NR4 7TJ, UK.

*Joint first authors

SI Figures

Figure S1. miRNA regulation of the transferome genes. Network of putative interactions between miRNAs (colourless nodes) and transferome genes whose products have a function in: development/cellular organisation (red), gene expression (orange), post-mating behaviour/response (green), response to stimulus (purple), metabolic process (yellow), protein processing (blue), unknown function (grey). Node size is directly proportional to the number of edges.

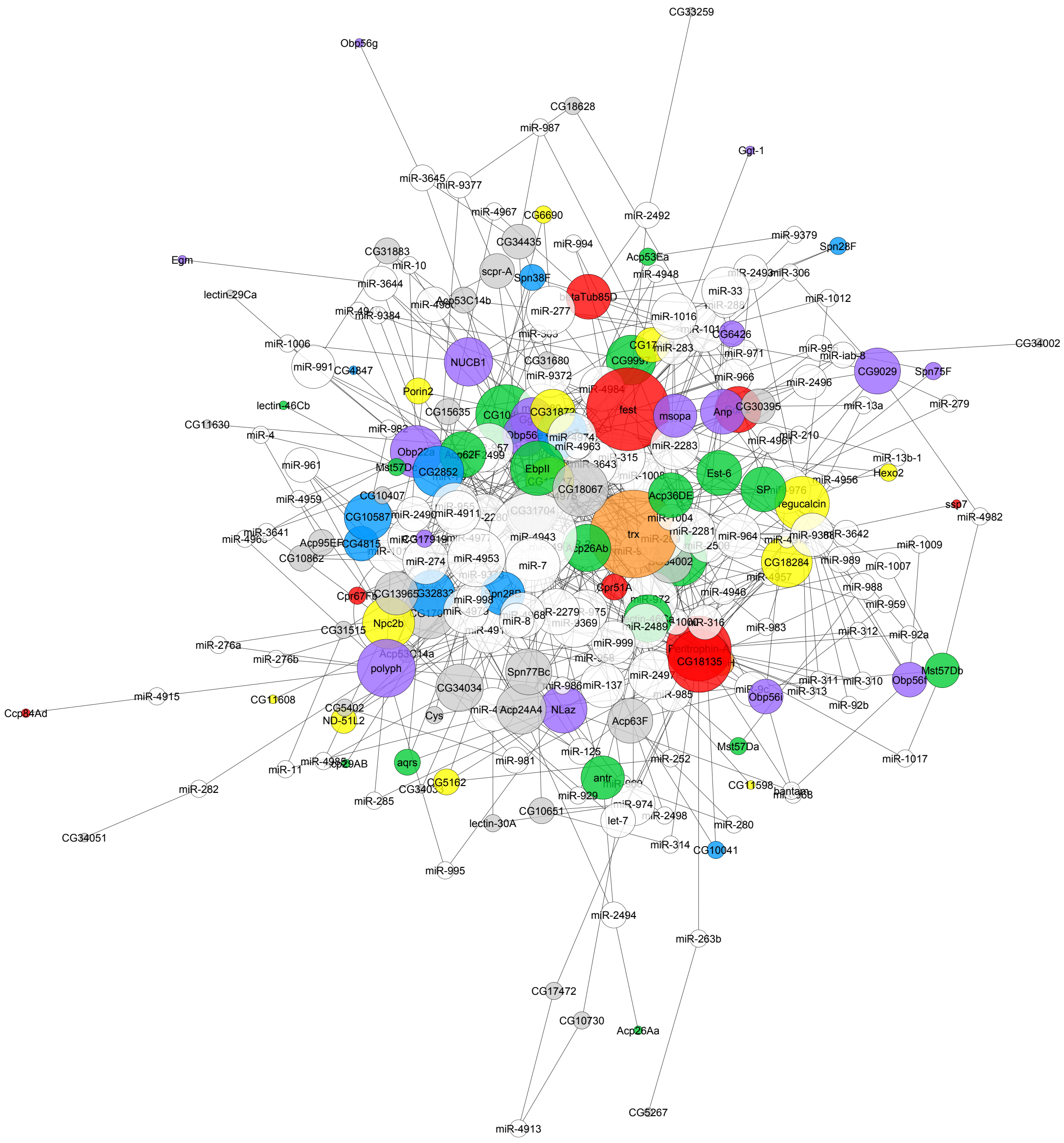
CG33259
Obp56g
CG18628
miR-987
Ggt-1
miR-3645
miR-9377
miR-4967
CG6690
miR-2492
miR-9379
CG31883
CG34435
miR-994
Spn28F
Egm
miR-10
Acp53Ea
scpr-A
miR-4948
miR-2493
miR-306
miR-3644
Spn38F
miR-33
lectin-29Ca
miR-9384
Acp53C14b
miR-277
miR-1012
miR-1016
CG6426
miR-1006
CG17
miR-283
CG34002
miR-991
CG4847
NUCB1
CG31680
CG9997
miR-971
CG9029
Spn75F
miR-9372
miR-966
miR-2496
Porin2
miR-4984
lectin-46Cb
CG15635
CG31872
fest
msopa
Anp
CG30395
miR-13a
miR-279
CG11630
miR-4963
miR-210
miR-4
miR-2283
miR-13b-1
miR-315
Hexo2
miR-961
Obp22a
CG2852
Ebpll
miR-3643
Est-6
Mst57Dc
CG18067
SP
regucalcin
miR-4956
ssp7
miR-4959
CG10407
Acp36DE
miR-4982
CG10587
miR-2490
miR-4911
CG31704
miR-1004
trx
miR-3641
miR-2281
miR-964
miR-3642
Acp95EF
CG4815
CG17919
miR-4943
Acp26Ab
miR-1009
CG10862
miR-274
miR-4953
miR-7
CG18284
miR-989
miR-1007
miR-4957
Cpr67Fb
CG13965
Cpr51A
miR-4946
miR-988
miR-998
miR-972
miR-959
miR-2279
miR-8
miR-316
miR-983
CG31515
Npc2b
miR-312
miR-92a
miR-276a
miR-2489
miR-999
Pertrophin-A
CG18135
miR-276b
polyph
Mst57Db
Spn77Bc
miR-137
miR-2497
miR-311
miR-310
Obp56f
CG34034
miR-986
miR-313
miR-4915
miR-985
Obp56i
miR-92b
Ccp84Ad
CG11608
CG5402
NLaz
ND-51L2
Cys
Acp24A4
Acp63F
Mst57Da
miR-1017
aqrs
miR-125
miR-252
miR-4985
Acp29AB
miR-981
miR-11
antr
CG11598
bantam
miR-282
CG5162
miR-929
miR-285
miR-974
CG10651
miR-2498
lectin-30A
let-7
miR-280
CG34051
miR-314
CG10041
miR-995
miR-2494
miR-263b
CG17472
CG10730
Acp26Aa
CG5267
miR-4913

Figure S2. Summary of the similarity (proportional identity) in pairwise comparisons of sets of UTRs potentially targeted by the same miRNAs. The similarity analysis was conducted where there were sets of at least 3 transcripts targeted by the same miRNA (the 87 such 3p/5p miRNAs potentially targeting >3 transcripts are shown on the X axis). Proportional identities (Y axis) are summarised by the median ±IQR similarity of all pairwise comparisons between transcripts targeted by the same miRNA. Overall, the median identity across all sets of transcripts oscillated in the 45-55% range, with the maximum identity exceeding 70% for only 11/87 sets. Hence for the majority of transcripts predicted to be targeted by the same miRNA, the similarity was < 60%, giving no evidence that they were paralogues. The inset shows an example weighted scatterplot for the 5 transcripts potentially targeted by miR-972-3p, calculated using Clustal Omega, reported as a proportion of the length of the shortest transcript in each case - the transcripts are presented in the same order on the X and Y axes, hence the diagonal values illustrate the comparison of each sequence against itself (100% similarity (proportional identity)).

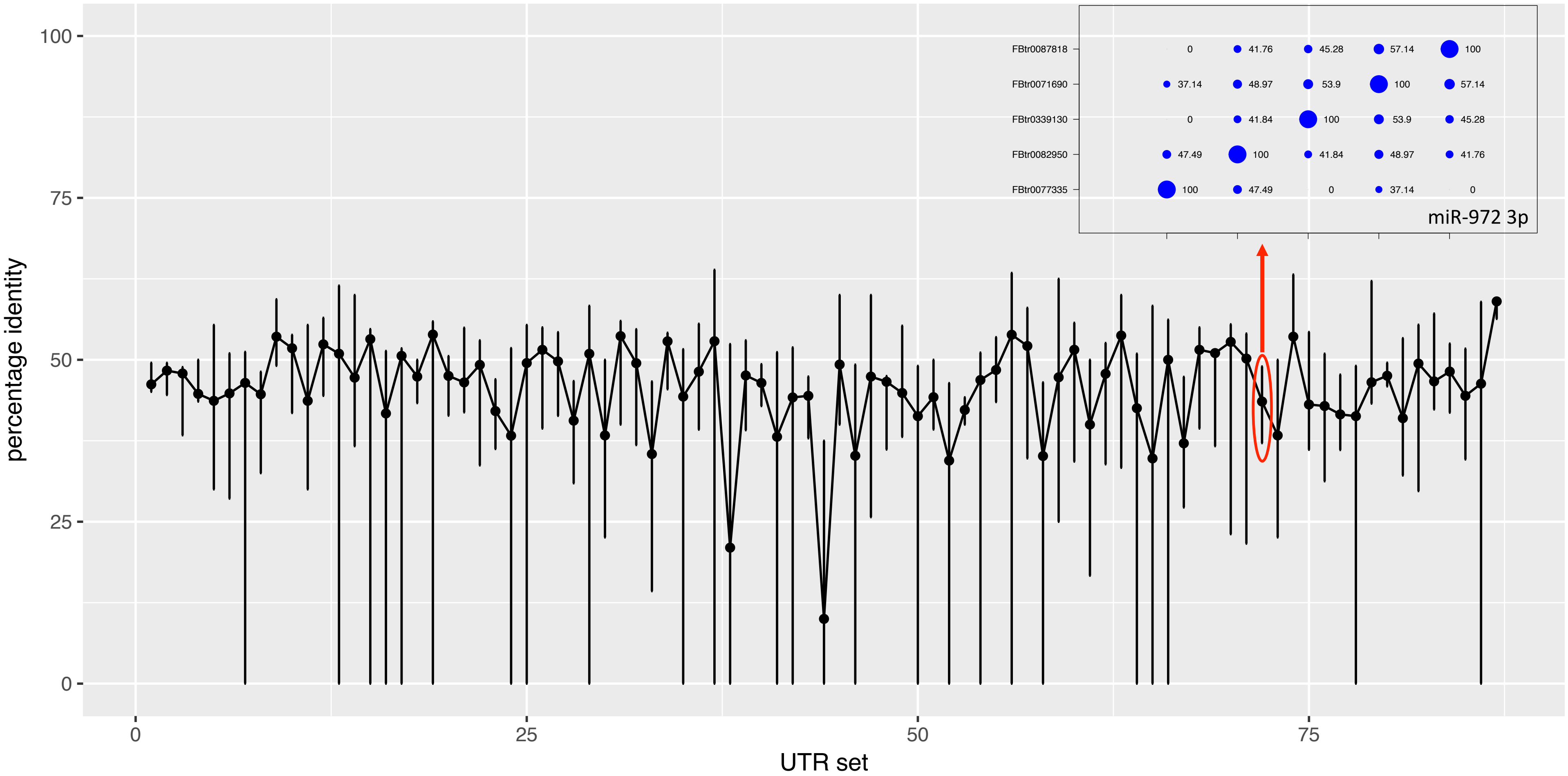
percentage identity
UTR set
miR-972 3p
FBtr0087818
FBtr0071690
FBtr0339130
FBtr0082950
FBtr0077335
0 41.76 45.28 57.14 100
37.14 48.97 53.9 100 57.14
0 41.84 100 53.9 45.28
47.49 100 41.84 48.97 41.76
100 47.49 0 37.14 0
